# Correlation between sleep problems and morning serum melatonin and ferritin levels in Japanese 5‐year‐old children with autism spectrum disorder

**DOI:** 10.1002/pcn5.70294

**Published:** 2026-02-10

**Authors:** Ai Terui, Manabu Saito, Asami Kuki, Shuji Shimoyama, Yui Sakamoto, Kazutaka Yoshida, Ayako Osato, Kazuhiko Nakamura

**Affiliations:** ^1^ Department of Neuropsychiatry, Graduate School of Medicine Hirosaki University Hirosaki Aomori Japan; ^2^ Department of Clinical Psychological Science, Graduate School of Health Sciences Hirosaki University Hirosaki Aomori Japan; ^3^ School of Medicine Hirosaki University Hirosaki Aomori Japan; ^4^ Postgraduate Clinical Training Center Jichi Medical University Hospital Tochigi Japan; ^5^ Department of Neurophysiology, Biomedical Research Center, Graduate School of Medicine Hirosaki University Hirosaki Aomori Japan

**Keywords:** 5‐year‐old children, autism spectrum disorder, ferritin, Japanese Sleep Questionnaire for Preschoolers (JSQP), melatonin

## Abstract

**Aim:**

Children with autism spectrum disorder (ASD) are more likely to have sleep problems. Few studies have investigated the relationship between sleep problems and blood melatonin and ferritin levels. The objective of this study was to determine the correlation between sleep problems and morning serum melatonin and ferritin levels, and the differences in serum melatonin and ferritin levels between children with ASD and those without ASD.

**Methods:**

Four years of data from population‐based 5‐year‐old checkups were referenced. Fifty‐five children were divided into the ASD group (*N* = 45) and the non‐ASD group (*N* = 10). Blood samples were collected at 8:30 a.m. The Japanese Sleep Questionnaire for Preschoolers (JSQP) was used to assess sleep problems. Correlation analysis, the Mann–Whitney *U* test, and multiple regression analysis were used.

**Results:**

In the ASD group, the score of Sleep habit was significantly correlated with the serum ferritin level (*ρ* = 0.496, *p* < 0.001). No significant regression equation was found. However, the partial correlation coefficient calculated indicated a significant value between the score of Insomnia or Circadian rhythm disorder and serum melatonin level (*β* = 0.502, *p* < 0.05), and the score of Sleep habit and the serum ferritin level (*β* = 0.546 *p* < 0.01). The serum ferritin level in the ASD group (23.48 ± 9.14 ng/mL) was significantly higher than in the non‐ASD group (14.84 ± 7.09 ng/mL) (*p* < 0.05).

**Conclusion:**

This study indicated that children with ASD were more likely to have some sleep problems and higher morning serum ferritin levels than those without ASD. Further research is recommended on the correlation between sleep problems and morning serum melatonin and ferritin levels.

## INTRODUCTION

### Importance of sleep for children

Sleep is one of the most important vital activities for humans, stabilizing not only many physiological functions but also higher brain functions and psychomotor functions. A meta‐review reported that sleep duration is consistently related to adiposity and emotional outcomes in children.^1^ A systematic review reported associations between sleep, behavior, and cognition as early as preschool years.^2^ One cohort study reported that irregular sleep routines at 5.8 years of age were associated with psychotic experiences at 12–13 years of age.^3^ A longitudinal study reported that short sleep duration and frequent nightly awakenings at 1.5 years of age predicted the development of depressive symptoms at 8 years of age.^4^

The National Sleep Foundation recommends 10–13 h of sleep for 5‐year‐old children.^5^ According to a survey conducted by the Japan Children's Health Association in 2010, 24.7% of 5–6‐year‐old Japanese children slept less than 9 hours.^6^ According to the Organization for Economic Co‐operation and Development (OECD), “Gender data portal 2021,” 15–64‐year‐old Japanese people sleep an average of 7 h and 22 min, the shortest among 33 countries in the world, which are mostly developed countries. The sleep of 3–6‐year‐old children in Asia, including Japan, differs from that in the West in that bedtime is significantly later, nighttime sleep is shorter, naps are more frequent, and sharing beds and rooms with parents is more common.^7^ In addition, Japanese children reported differences in sleep compared with their Chinese counterparts in Asia, including higher scores on the CSHQ (Children's Sleep Habits Questionnaire) bedtime resistance, earlier bedtime and wake‐up times, and shorter total sleep times.^8^

### Sleep in ASD

Autism spectrum disorder (ASD) is a neurodevelopmental disorder (NDD) characterized by impaired social communication and limited and repetitive behaviors.^9^ The reported prevalence of ASD varies, but in 2020, the United States of America reported 2.76% of 8‐year‐old children. This study also revealed that the percentage of children with ASD is increasing.^10^ Children with ASD are frequently complicated by other NDDs, such as attention‐deficit/hyperactivity disorder (ADHD), intellectual disability (ID), and developmental coordination disorder (DCD).^11^

The reported prevalence of sleep problems in children with ASD varies, but these children are more likely to have sleep problems than typically developing (TD) children are, with a review in 2022 reporting that 37% to 93% (71.41 ± 15.92) of children with ASD have sleep problems.^12^ Kuki et al. reported that the prevalence of sleep disorders in 5‐year‐old children was 18% of the total population in one city, whereas it was significantly higher in children with ASD, at 50.4%.^13^

ADHD is one of the NDDs that children with ASD are prone to have comorbidity with. It has been reported that children with ADHD experience more sleep problems, such as daytime sleepiness, more movements during sleep, and higher apnea–hypopnea indices, than children without ADHD.^14^

### Melatonin

Hormones involved in sleep include orexin, which maintains wakefulness; GABA (Gamma‐Amino Butyric Acid), which is known as an inhibitory neurotransmitter; and melatonin, which is involved in sleep induction and circadian rhythm regulation. Only melatonin is used therapeutically for sleep in children in Japan.

Melatonin (N‐acetyl‐5‐methoxytryptamine) is a hormone produced from serotonin, mainly in the pineal gland. Melatonin, produced in the pineal gland, produces sleep‐inducing effects by binding to MT1 receptors expressed in the suprachiasmatic nucleus and rhythm‐regulating effects by binding to MT2 receptors. Normally, melatonin is inhibited by light and has a cycle in which its concentration peaks during the night, and decreases during the day, and is involved in sleep induction and rhythm regulation.^15^ It is known that exposure to bright light at night shifts melatonin secretion to the morning.^16^, ^17^ Melatonin can be measured in blood (plasma and serum), saliva, and urinary metabolites. There are not many previous studies measuring morning melatonin levels. Several studies have shown that morning blood melatonin levels are lower in individuals with ASD than in individuals with TD,^18^, ^19^, ^20^ whereas others have reported that morning serum melatonin levels and urinary metabolite excretion of melatonin metabolites are higher in individuals with ASD than in individuals with TD;^21^, ^22^ however, the conclusions have not been reached.

### Ferritin

Iron is important for homeostasis, including hemoglobin formation, antioxidant activity, and gene repair. Iron is a cofactor in the production of brain transmitters such as dopamine and serotonin, and is also important as a component of dopamine D1 and D2 receptors.^23^ Iron deficiency can severely impair central nervous system development. Previous studies have shown a link between iron and mental and motor function and intellectual development.^24^

Ferritin is a protein widely distributed in liver parenchymal cells and the liver, spleen, bone marrow, etc. Ferritin serves as a form of iron storage in the body and can be measured as serum ferritin. The serum ferritin level is helpful in the diagnosis of iron deficiency because it falls earlier than the serum iron level in individuals with iron deficiency, shows less circadian variation than the serum iron level does, and correlates directly with the body's iron stores.

### Objective and significance

Very few biological studies have been conducted on Japanese 5year‐olds using scales tailored to Japanese sleep habits. Therefore, the objective of this study was to determine whether morning serum melatonin and ferritin levels are correlated with sleep problems and to determine whether differences in morning serum melatonin and ferritin levels exist between 5‐year‐old children with ASD and those without ASD.

The results of this study may allow us to examine the usefulness of replacement therapy for melatonin and ferritin and to consider appropriate treatment modalities for sleep problems. If information on children's sleep problems can be obtained with a single blood collection in the morning, it would be less invasive and very meaningful.

## METHOD

### Sampling

In this study, data from the population‐based Hirosaki 5‐Year‐Old Developmental Health Cohort Study (HFC Study) database of children who participated in the second phase of checkups in 2014, 2015, 2018, and 2019, when the Japanese Sleep Questionnaire for Preschoolers (JSQP) was conducted, and whose parents consented to blood collection for their children, were referenced.

### Discussion of the HFC study database

The HFC study is a cohort study using infant health examinations that has been conducted in cooperation with Hirosaki City and Hirosaki University since 2013.^11^ The purpose of this study is the early detection of NDDs and to provide early support. The study population included all 5‐year‐old children living in Hirosaki City, with 1200–1300 children per year. Hirosaki City is located in Aomori Prefecture, Japan, with a population of 160,129 and 70,910 households; the population under 15 years old was 16,269, and the population of 5‐year‐old children was 1034 in May 2024.

**Figure 1:**
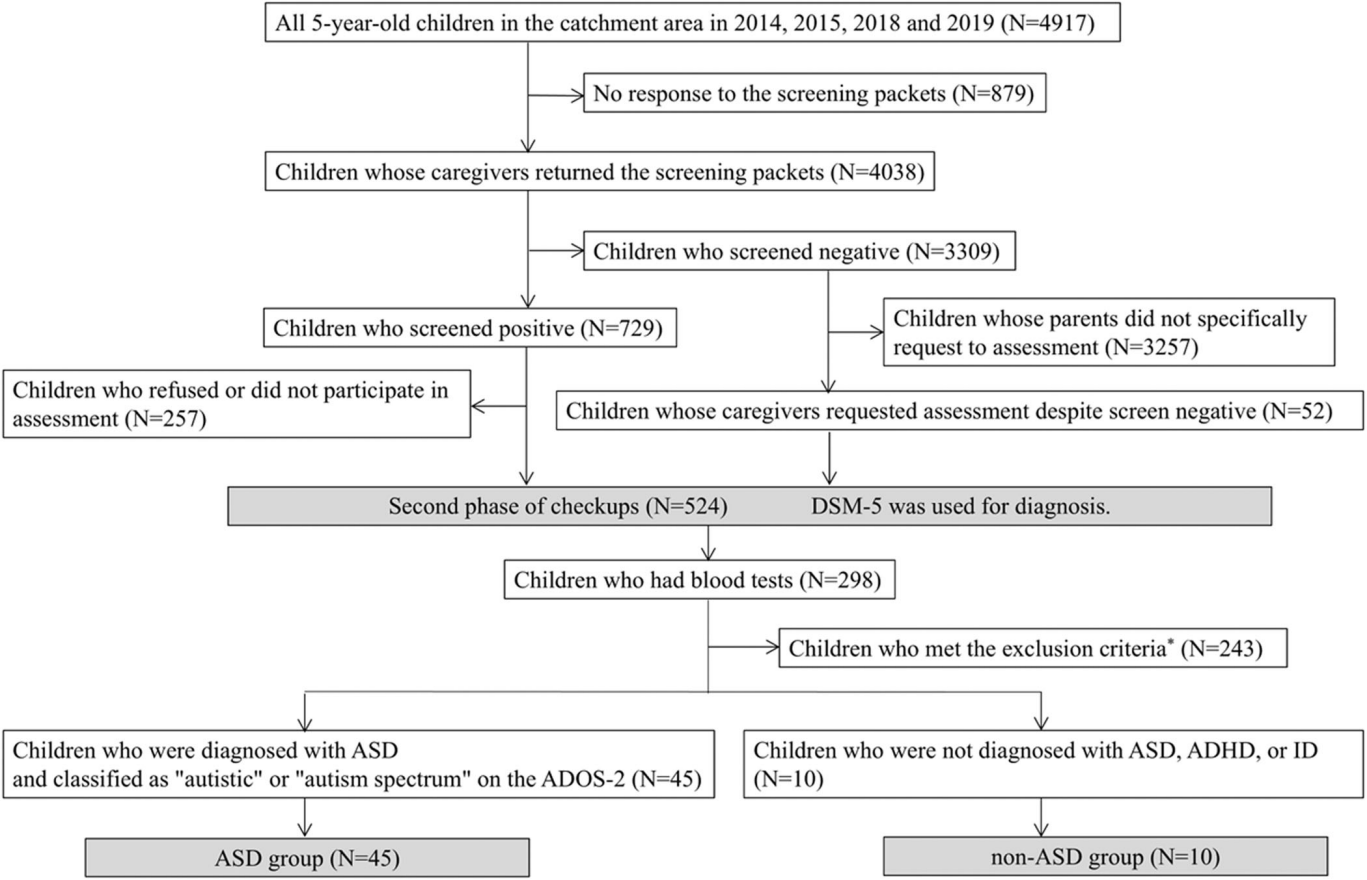
The flow chart of subjects extracted from the population‐based 5‐year‐old checkups. DSM‐5: Diagnostic and Statistical Manual of Mental Disorders, 5th edition. ASD: autism spectrum disorder. ADHD: attention‐deficit/hyperactivity disorder. ID: intellectual disability. ADOS‐2: Autism Diagnostic Observation Schedule, second edition exclusion criteria* did not meet the criteria of the ASD group and the non‐ASD group, whose serum was less than a measurable amount (500 μL) of melatonin, be taking iron medicine.

The HFC study was conducted in two phases (Figure 1). Details of each phase of the HFC study are shown in the appendices [A] and [B]. The first phase involved screening for NDDs that target all 5‐year‐old children living in Hirosaki City. The second phase targeted children who screened positive and a few children who were screening negative but whose caregivers requested an assessment. The purpose of the second phase was to diagnose NDDs by performing several developmental tests. In the second phase of the check‐up, parents were encouraged to have their children's blood tested. At least half of the parents consented to a blood test for their children, and their children had their blood collected at a later date at the university hospital. The blood tests included mandatory tests for thyroid hormones and somatomedin, and consent was obtained for the other tests to be used for the study. Children were diagnosed with ASD, ADHD, ID, motor disorders (DCD, Tic disorder), communication disorders (language disorder, speech disorder, childhood‐onset fluency disorder), and selective mutism according to the criteria of the Diagnostic and Statistical Manual of Mental Disorders, 5th Edition (DSM‐5). Some children are diagnosed with multiple coexisting NDDs.

### Setting

There were 298 children who had blood tests performed during the four‐year period covered by this study. In this study, children diagnosed with ASD by the DSM‐5 and classified as “autism” or “autism spectrum” by the Autism Diagnostic Observation Schedule, Second Edition (ADOS‐2) at the second phase of the checkup were defined as the “ASD group,” and children who were not diagnosed with ASD, ADHD, or ID at the second phase of the checkup were defined as the “non‐ASD group.” Two hundred forty‐three children were excluded, who did not meet the criteria of the two groups, whose serum was less than a measurable amount (500 μL) of melatonin, and who were taking iron medicine. The final subject of this study was 55 children, 45 in the ASD group and 10 in the non‐ASD group. All 10 children in the non‐ASD group were negative at the screening of the first phase. In the ASD group, we decided not to exclude children with comorbidities such as ADHD, ID, DCD, and communication disorders (language disorders, speech disorders, and childhood‐onset fluency disorders). The non‐ASD group did not include children with comorbid ADHD and ID but did include children with comorbid DCD and communication disorders (Table 1). In this study, it was impossible to increase the sample size further because no more children met the criteria for the ASD group or the non‐ASD group.

**Table 1:** Number of children diagnosed with NDDs in the 2 groups.

	ASD group	non‐ASD group
ASD	45/45	0/10
ADHD	20/45	0/10
ID	5/45	0/10
Motor Disorders (DCD, Tic disorder)	23/45	2/10
Communication disorders (Language Disorder, Speech Disorder, Childhood‐Onset Fluency Disorder)	10/45	1/10
Selective mutism	2/45	0/10

The children in this study did not take melatonin orally because melatonin preparations were not approved in Japan until 2020.

### Ingredients

#### Blood collection methods

Blood was collected from fasted children within two months of the second phase of the checkup into a nonadditive blood collection tube from a vein in the forearm at approximately 8:30 a.m. using the usual technique. The blood samples were immediately centrifuged at 3000 rpm for 10 min to separate the serum. The serum was stored frozen at −80°C without freeze–thaw cycles.

#### Melatonin measurement methods

Serum melatonin levels were measured using an ARG80461 Human Melatonin ELISA (Enzyme‐Linked Immunosorbent Assay) Kit (arigo Biolaboratories Corp.) based on the competitive enzyme immunoassay technique. The standard range for this kit is 3–270 pg/ml. Five hundred microliters of serum was used for our measurements. The absorbance at 405 nm was measured using a microplate reader (MULTISKAN FC, ThermoFisher Scientific, Waltham, MA). Measurements were performed in duplicate, and the average value was used as the result.

#### Ferritin measurement methods

Serum ferritin levels were measured via the LUMIPULSE Presto Ferritin reagent (Fujirebio) based on the CLEIA assay (two‐step sandwich method) principle. A total of 400 µL of serum was used for our measurements. A Lumipulse Presto II (Fujirebio) was used as the measuring instrument.^25^

#### JSQP

The JSQP, a Japanese culture‐based sleep questionnaire, was used to assess sleep habits. JSQP was developed in 2010 by researchers at the Department of Pediatrics, Osaka University. The caregiver observation‐based questionnaire consists of 39 questions on a 6‐point strength Likert scale (not at all true, not true, somewhat not true, somewhat true, true, and very true), with 10 subitems (Ⅰ: RLS sensory, Ⅱ: RLS motor, Ⅲ: OSAS (obstructive sleep apnea syndrome), Ⅳ: parasomnia, Ⅴ: insomnia or circadian rhythm disorder, Ⅵ: morning symptoms, Ⅶ: daytime excessive sleepiness, Ⅷ: daytime behaviors, Ⅸ: sleep habit, Ⅹ: insufficient sleep).^26^ The questions included two reversal items to ensure consistency of the responses, which were recalculated prior to tabulation. Details of the questions of JSQP are shown in the appendices [C].2.5 Statistical analysis.

The data were analyzed using SPSS for Windows statistical package version 28 (SPSS Inc).

To examine how sleep problems in 5‐year‐old children are associated with serum melatonin and ferritin levels, nonparametric Spearman's correlation analysis was conducted. Furthermore, multiple regression analysis with forced entry methods was conducted with serum melatonin and ferritin levels as the dependent variables and JSQP subitems and total score of ADHD‐RS as the independent variables. The reason for including the total score of ADHD‐RS as the independent variable was that the ASD group included children with comorbid ADHD, and children with ADHD tend to have more sleep problems, necessitating control. The significance level was set at *p* < 0.05.

To compare differences in morning serum melatonin and ferritin levels, and the scores of the JSQP subitems between children with ASD and those without ASD, a nonparametric Mann–Whitney *U* test was used. The significance level was set at *p* < 0.05.

## RESULTS

### Characteristics of the two groups

There were no significant differences in age (months), sex, or total IQ between the ASD group and the non‐ASD group. On the other hand, the total scores on the ASSQ (Autism Spectrum Screening Questionnaire), ADHD‐RS (ADHD‐Rating Scale Ⅳ), and SDQ (Strengths and Difficulties Questionnaire) were all significantly higher in the ASD group. The ADOS‐2 comparison score for the ASD group was 6.49 ± 1.70 (moderate level) (Table 2).

**Table 2:** Developmental features of the two groups.

	ASD group	non‐ASD group	*p* value
Age (months)	64.11 ± 1.98	64.50 ± 2.17	n.s.
Sex (percentage of boys)	34/45 (75.6%)	8/10 (80.0%)	n.s.
IQ	88.98 ± 13.82	94.20 ± 14.92	n.s.
Total score of ASSQ	14.78 ± 9.09	5.80 ± 5.39	<0.01
Total score of ADHD‐RS	20.53 ± 11.33	5.10 ± 3.41	<0.001
Total score of SDQ	15.60 ± 4.70	8.70 ± 2.16	<0.001
ADOS‐2 comparison score	6.49 ± 1.70	－	－

*Note*: The nonparametric Mann–Whitney *U* test was used to determine differences in age in months, IQ, and total scores on the ASSQ, ADHD‐RS, and SDQ. The significance level was set at *p* < 0.05. Pearson's chi‐square test was used to determine differences in sex. The significance level was set at *p* < 0.05.

### Nonparametric correlation analysis of each group

In the ASD group, the score of JSQP IX: sleep habit was significantly positively correlated with the serum ferritin level (*ρ* = 0.496, *p* < 0.001) (Table 3). In the non‐ASD group, there was no correlation. In both groups, there was no correlation between the serum melatonin levels and the serum ferritin levels.

**Table 3:** Correlations between serum melatonin/ferritin levels and JSQP subitem scores.

JSQP subitems		Ferritin		Melatonin	
		non‐ASD group	ASD group	non‐ASD group	ASD group
Ⅰ.	RLS sensory	−0.472	0.000	−0.225	−0.084
Ⅱ.	RLS motor	−0.112	0.197	−0.199	0.101
III.	OSAS	0.502	0.066	0.061	0.145
IV.	Parasomnias	−0.107	−0.021	−0.289	−0.024
V.	Insomnia or circadian rhythm disorder	0.434	0.031	0.281	0.117
VI.	Morning symptoms	−0.358	0.278	0.500	0.081
VII.	Daytime excessive sleepiness	0.188	0.073	−0.194	0.163
VIII.	Daytime behaviors	−0.372	0.069	0.341	0.000
IX.	Sleep habit	0.323	**0.496** ***	0.311	−0.105
X.	Insufficient sleep	−0.095	0.040	0.432	−0.045

*Note*: Each number in the table represents Spearman's correlation coefficient.

Bold indicates *p*‐values less than 0.05.

*** *p* < 0.001

### Multiple regression analysis of each group

**Figure 2:**
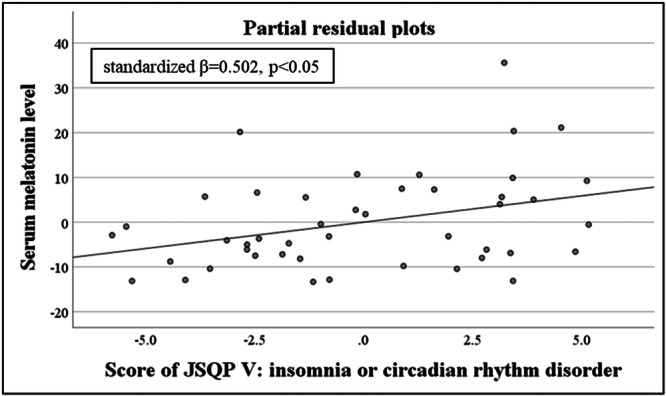
Partial residual plots between the serum melatonin level and JSQP V score: insomnia or circadian rhythm disorder; the vertical axis shows the residuals of the mean serum melatonin level, and the horizontal axis shows the predicted JSQP V score: Insomnia or circadian rhythm disorder. JSQP: Japanese Sleep Questionnaire for Preschoolers.

**Figure 3:**
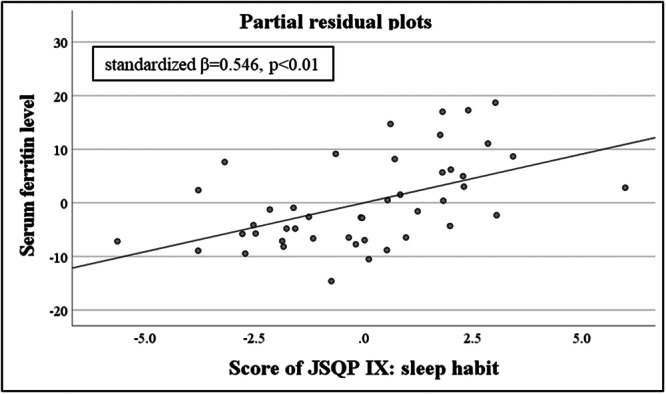
Partial residual plots between the serum ferritin level and JSQP Ⅸ score: sleep habit; the vertical axis shows the residuals of the mean serum ferritin level, and the horizontal axis shows the predicted score of JSQP Ⅸ: Sleep habit. JSQP: Japanese Sleep Questionnaire for Preschoolers.

In the ASD group, when the dependent variable is serum melatonin level, a significant regression equation was not found (adjusted *R* ^2^ = −0.033, *p* = no significant). However, the partial correlation coefficient calculated using the forced entry method indicated a significant value between the score of JSQP V: insomnia or circadian rhythm disorder and serum melatonin level (standardized *β* = 0.502, *p* < 0.05) (Figure 2) (Table 4). Similarly, when the dependent variable is serum ferritin level, a significant regression equation was not found (adjusted *R* ^2^ = −0.199, *p* = no significant). However, the partial correlation coefficient calculated using the forced entry method indicated a significant value between the score of JSQP IX: sleep habit and the serum ferritin level (standardized *β* = 0.546, *p* < 0.01) (Figure 3) (Table 4). In the non‐ASD group, results could not be calculated because the sample size of the non‐ASD group was too small.

**Table 4:** Partial correlation coefficients between sleep problems and serum melatonin and ferritin levels in the ASD group.

Dependent variables	Independent variables					
	Ferritin			Melatonin		
	Standardized *β*	*t* value	*p* value	Standardized *β*	*t* value	*p* value
JSQP Ⅰ. RLS sensory	−0.093	−0.556	0.582	−0.169	−0.891	0.379
JSQP Ⅱ. RLS motor	0.065	0.391	0.698	0.187	0.993	0.328
JSQP Ⅲ. OSAS	0.073	0.443	0.66	0.142	0.763	0.451
JSQP IV. Parasomnias	0.029	0.176	0.861	−0.094	−0.504	0.617
JSQP V. Insomnia or circadian rhythm disorder	−0.380	−1.832	0.076	**0.502**	**2.128**	**0.041**
JSQP VI. Morning symptoms	0.313	1.723	0.094	0.111	0.536	0.596
JSQP VII. Daytime excessive sleepiness	0.242	1.286	0.207	−0.010	−0.045	0.965
JSQP VIII. Daytime behaviors	−0.080	−0.454	0.653	−0.203	−1.008	0.321
JSQP IX. Sleep habit	**0.546**	**3.432**	**0.002**	−0.211	−1.170	0.251
JSQP X. Insufficient sleep	0.252	1.495	0.144	−0.328	−1.708	0.097
Total score of ADHD‐RS	0.132	0.826	0.415	0.046	0.256	0.799

*Note*: Bold indicates *p*‐values less than 0.05.

### Morning serum melatonin levels

**Figure 4:**
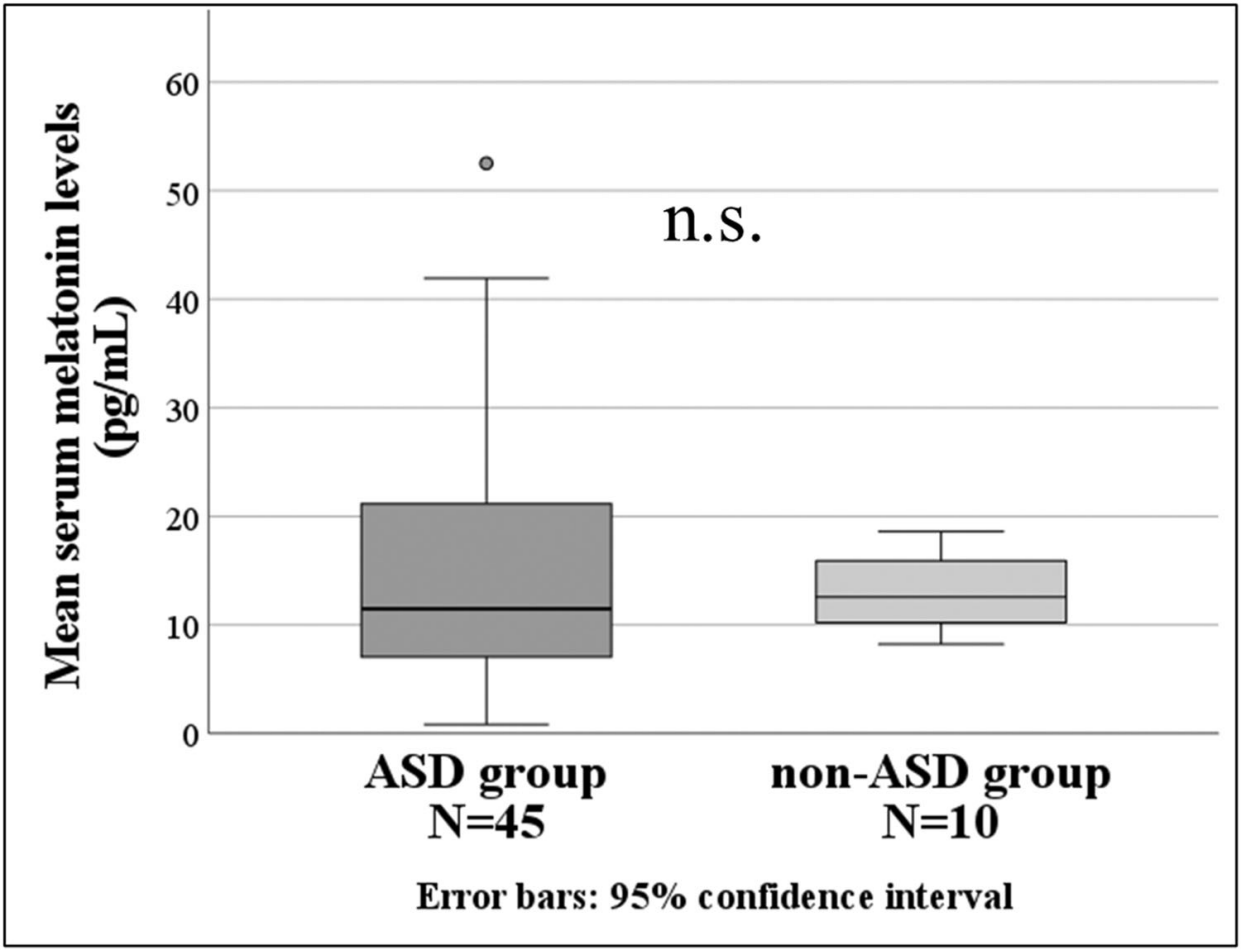
Morning serum melatonin levels; the vertical axis shows the serum melatonin levels, and the horizontal axis shows the ASD group and non‐ASD group. ASD, autism spectrum disorder.

There were no significant differences in morning serum melatonin levels between the ASD group (15.37 ± 11.35 pg/mL) and the non‐ASD group (13.34 ± 3.65 pg/mL) (Figure 4).

### Morning serum ferritin levels

**Figure 5:**
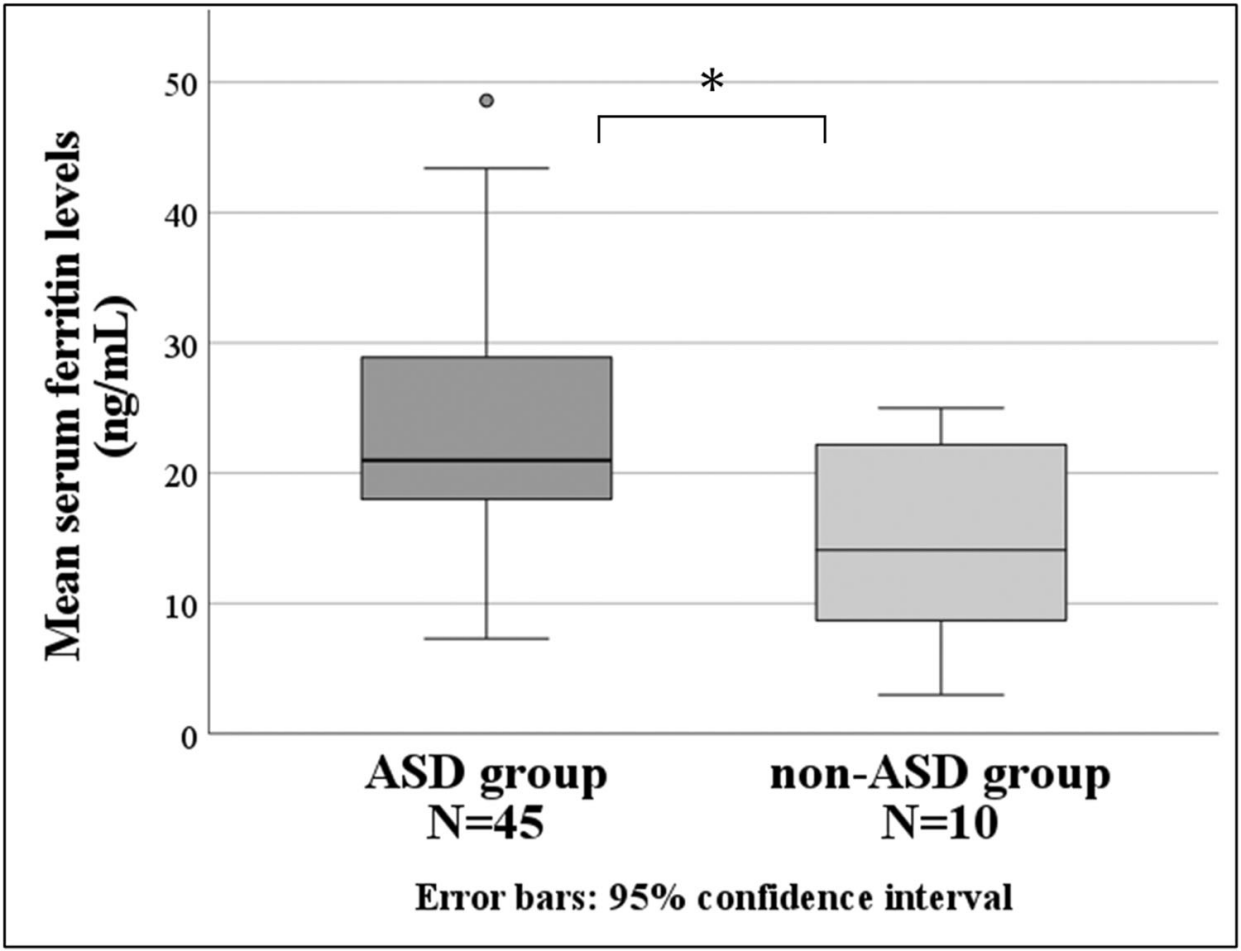
Morning serum ferritin levels; the vertical axis shows the serum ferritin levels, and the horizontal axis shows the ASD group and non‐ASD group. *p < 0.05. ASD, autism spectrum disorder.

The morning serum ferritin level was significantly higher in the ASD group (23.48 ± 9.14 ng/mL) than in the non‐ASD group (14.84 ± 7.09 ng/mL) (*p* < 0.05) (Figure 5).

### JSQP subitems

There were no significant differences in the mean JSQP total score between the ASD group (83.43 ± 17.26) and the non‐ASD group (72.40 ± 11.72).

**Figure 6:**
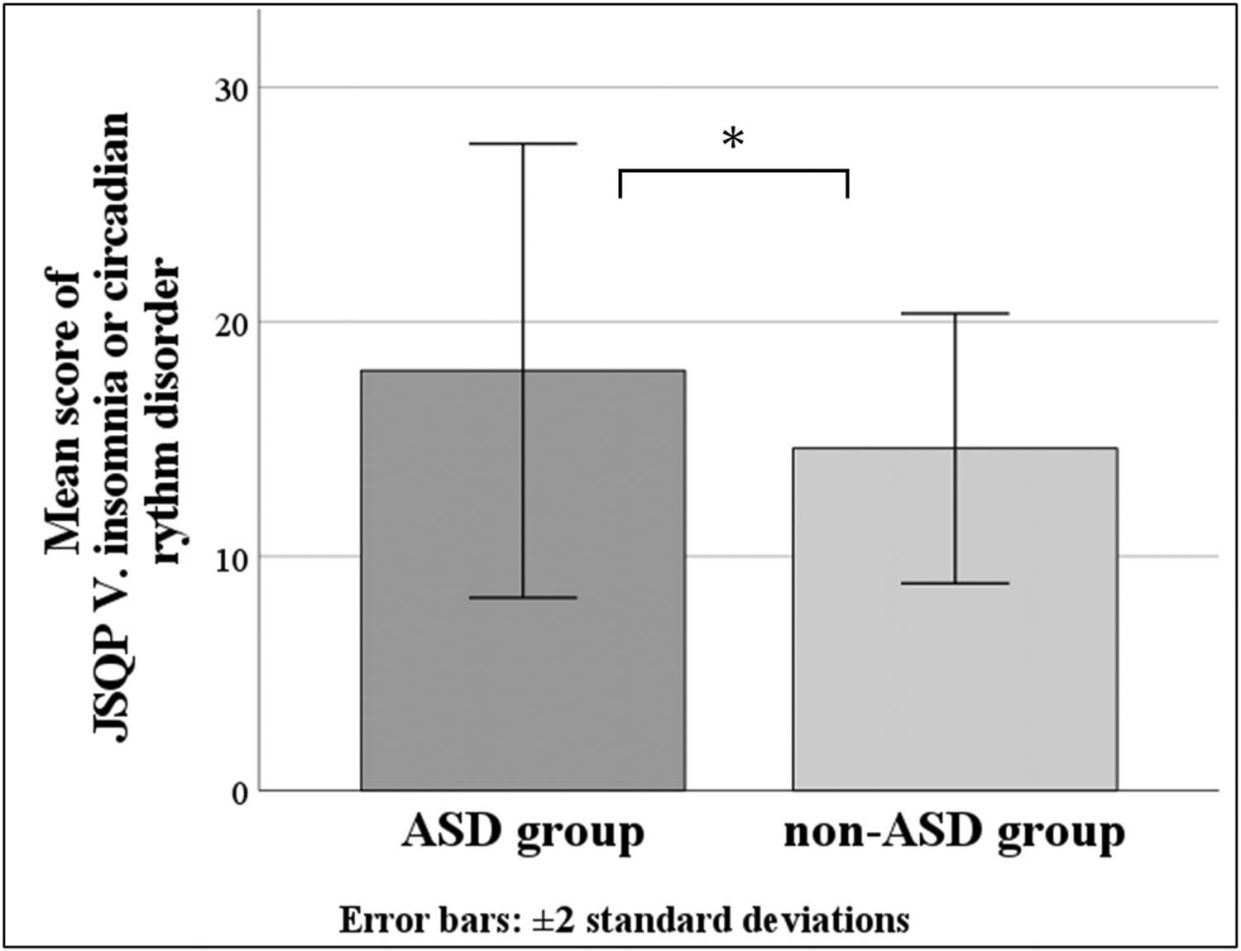
Mean JSQP V score: insomnia or circadian rhythm disorder; the vertical axis shows the JSQP V score: insomnia or circadian rhythm disorder, and the horizontal axis shows the ASD group and non‐ASD group. *p < 0.05. ASD, autism spectrum disorder; JSQP, Japanese Sleep Questionnaire for Preschoolers.

**Figure 7:**
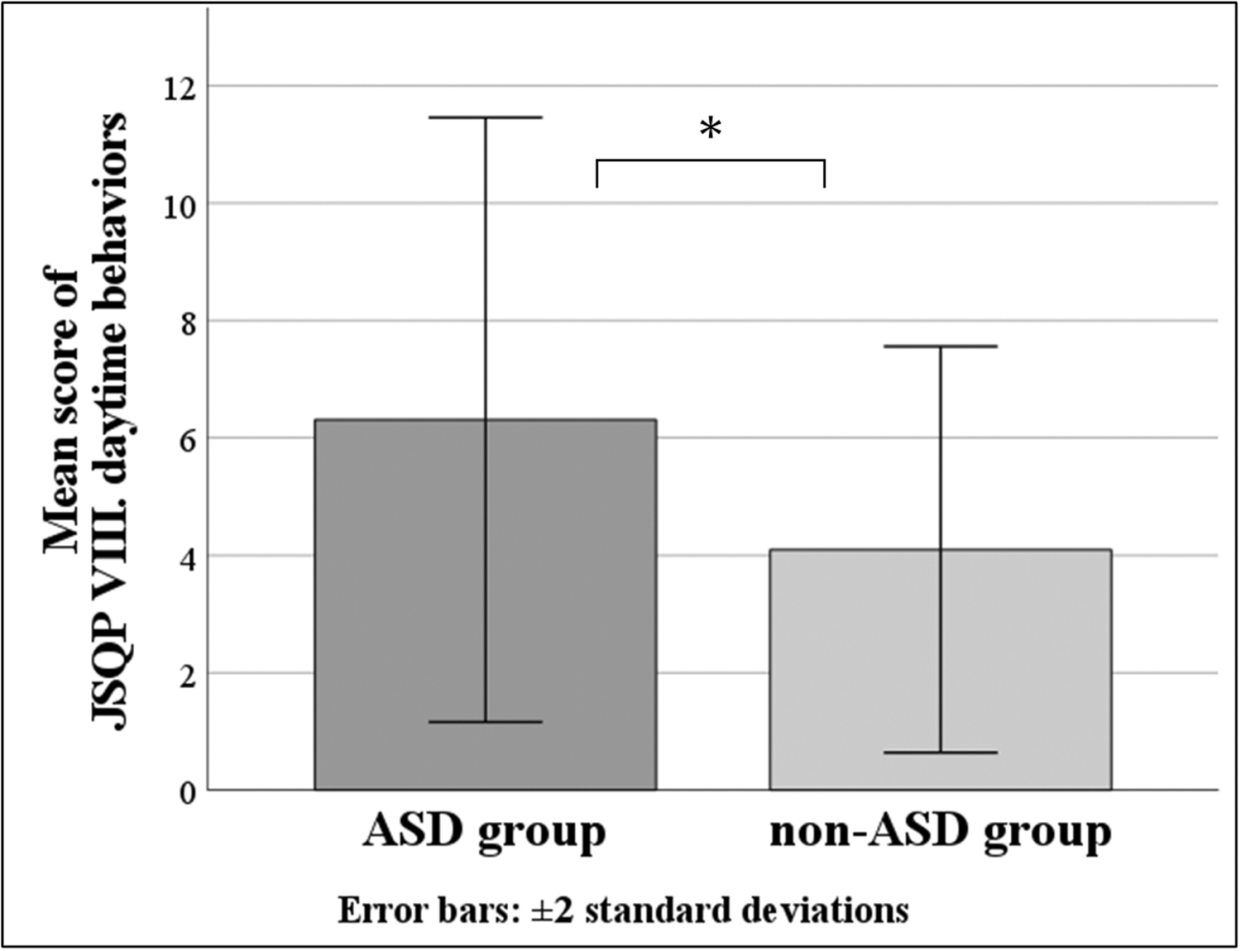
Mean JSQP VIII score: daytime behaviors; the vertical axis shows the score of VIII daytime behaviors, and the horizontal axis shows the ASD group and non‐ASD group. *p < 0.05. ASD, autism spectrum disorder; JSQP, Japanese Sleep Questionnaire for Preschoolers.

The mean score of the JSQP subitem V: insomnia or circadian rhythm disorder was significantly higher in the ASD group (17.92 ± 4.84) than in the non‐ASD group (14.60 ± 2.88) (*p* < 0.05) (Figure 6), and the mean score of the JSQP subitem VIII: daytime behaviors, was significantly higher in the ASD group (6.31 ± 2.58) than in the non‐ASD group (4.10 ± 1.73) (*p* < 0.05) (Figure 7). Other JSQP subitem scores were not significantly different between the two groups (Table 5).

**Table 5:** Mean scores of the JSQP subitems.

		ASD group	Non‐ASD group	*p* value
Ⅰ:	RLS sensory	4.07 ± 1.59	3.50 ± 0.97	n.s.
Ⅱ:	RLS motor	2.79 ± 1.62	2.50 ± 1.27	n.s.
III:	OSAS	17.44 ± 6.27	15.60 ± 3.66	n.s.
IV:	Parasomnias	7.44 ± 3.57	6.90 ± 2.18	n.s.
V:	Insomnia or circadian rhythm disorder	17.92 ± 4.84	14.60 ± 2.88	<0.05
VI:	Morning symptoms	9.07 ± 3.95	7.50 ± 3.38	n.s.
VII:	Daytime excessive sleepiness	5.31 ± 2.18	4.90 ± 1.97	n.s.
VIII:	Daytime behaviors	6.31 ± 2.58	4.10 ± 1.73	<0.05
IX:	Sleep habit	7.98 ± 2.73	7.90 ± 2.51	n.s.
X:	Insufficient sleep	5.11 ± 2.40	4.90 ± 3.00	n.s.

*Note*: The nonparametric Mann–Whitney *U* test was used to determine differences in the scores of the JSQP subitems between the ASD group and the non‐ASD group. The significance level was set at *p* < 0.05.

## DISCUSSION

### Correlations between serum ferritin levels and sleep problems

JSQP Ⅸ: sleep habits consisted of two questions, “sleeps without being tucked in” and “goes to bed on his or her own,” asking whether children sleep spontaneously. Morning serum ferritin levels and sleeping spontaneously in children with ASD might be related, but verification with a larger sample size is recommended. There are no previous studies reporting a relation between serum ferritin levels and sleeping spontaneously. If it could be that higher morning serum ferritin levels were related to being less likely to sleep spontaneously in children with ASD, these are two possibilities to consider. First, there may be an underlying association between high serum ferritin levels and difficulty sleeping spontaneously in children with ASD. Second, microinflammation may be undetected in children with difficulty sleeping spontaneously. Although the children did not have a fever on the morning of the blood test, they might have had minor physical problems, such as the common cold, without a fever. It would be helpful to examine other inflammatory markers.

### Morning serum ferritin levels

Most hypoferritinemia is known to be caused by iron deficiency. Serum ferritin, on the other hand, is elevated in malignancy, liver injury, myocardial infarction, infection, inflammation, etc., independent of iron stores.^27^, ^28^ Unlike melatonin, blood ferritin levels fluctuate little during the day.

Several previous studies have reported low blood ferritin levels in children with ASD, which is thought to be due in part to low iron intake due to an unbalanced diet. A previous study reported that 24.1% of 3–16‐year‐old children with ASD were iron deficient.^29^ However, a meta‐analysis revealed no difference in serum ferritin levels between children with ASD and those without ASD.^30^ The results of this study contradict this meta‐analysis. It should be noted that both the ASD and non‐ASD groups had low serum ferritin levels. In 2020, in apparently healthy children aged 5–10 years, the WHO recommended a cutoff value for iron deficiency as a serum ferritin concentration <15 ng/mL, and for the risk of iron overload as a serum ferritin concentration >150 ng/mL in females and >200 ng/mL in males.^31^ According to this cutoff value, the mean serum ferritin level of the non‐ASD group in this study was in the iron deficiency range, and the mean serum ferritin level of the ASD group was not high. The range of serum ferritin levels in the non‐ASD group ranged from 3.0 to 25.0 ng/mL. The outliers may have had a significant effect on the results because of the small sample size. Children with suspected iron deficiency were referred to pediatricians for closer examination. Investigating dietary iron intake in children may be helpful.

### Correlations between serum melatonin levels and sleep problems

Morning serum melatonin levels and insomnia or circadian rhythm disorders in children with ASD might be related, but verification with a larger sample size is recommended. If it could to be that higher morning serum melatonin levels were related with more insomnia or circadian rhythm disorders in children with ASD, several patterns may serve as mechanisms underlying elevated morning serum melatonin levels in children with insomnia or circadian rhythm disorder, such as a delayed phase of the rhythm of serum melatonin, a higher overall serum melatonin level, and a shifted amplitude of serum melatonin levels in children with insomnia or circadian rhythm disorder. Although few previous studies have examined the rhythms of melatonin increase and decrease in individuals with ASD, several reports have shown that the amplitude of circadian variation, which is normally lower levels during the day and higher levels at night, is smaller in individuals with ASD than in individuals with TD or that the phase is reversed, with lower levels during the day and higher levels at night^32^, ^33^, ^34^

**Figure 8:**
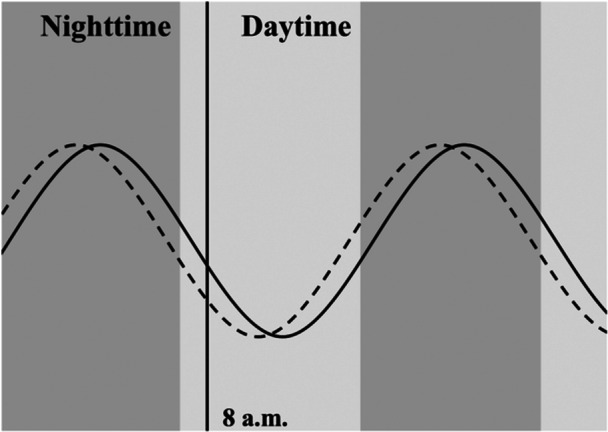
Possible pattern of melatonin increase and decrease; The possible melatonin rhythm of children without insomnia or circadian rhythm disorder is shown as dotted lines, and the possible melatonin rhythm of children with insomnia or circadian rhythm disorder is shown as solid lines.

Considering items of the JSQP V: insomnia or circadian rhythm disorder, such as “late for nursery school or kindergarten due to waking up late,” “goes to bed after 10:00 PM,” “day to night reversal,” a delayed phase of the rhythm of serum melatonin shown in Figure 8 may be plausible. An appropriate reduction in morning serum melatonin levels may lead to a decrease in insomnia or circadian rhythm disorders. Possible ways to lower morning serum melatonin levels include exposure to light in the morning and night, and melatonin administration to regulate the melatonin increase and decrease rhythm. To examine the difference in the rhythm of melatonin increase and decrease in greater detail, it is necessary to examine the melatonin level by collecting multiple samples throughout the day. Multiple measurements of melatonin level can be easily performed at home using non‐invasive salivary measurement kits.

### Morning serum melatonin levels

Several previous studies have shown that average daily blood melatonin levels are lower in individuals with ASD than in individuals with TD. In individuals with ASD, disruption of the melatonin‐producing pathway, the serotonin‐N‐acetylserotonin‐melatonin pathway, and its heritability have been suggested.^18^, ^35^, ^36^

Because of the small sample size, this study may be unable to state the difference in serum melatonin levels.

### JSQP subitems

In this study, there was no significant difference in the mean JSQP total score between the ASD group and the non‐ASD group. This contradicts the findings of previous studies, which reported that sleep problems were more prevalent in children with ASD than in children without ASD. The small sample size of this study may have affected the results.

In this study, children in the ASD group experienced more sleep problems related to insomnia or circadian rhythm disorders and daytime behaviors than the non‐ASD group. In a previous study conducted in China with 2–7‐year‐old children, the prevalence of sleep disorders in children with ASD was significantly higher than that in TD children (67.4% vs. 51%, *p* < 0.01), and children with ASD were more likely to have problems with bedtime resistance, sleep anxiety, delayed sleep onset, and daytime sleepiness at the CSHQ.^37^ Although the ASD group in this study experienced more insomnia or circadian rhythm disorders, these questions can be synonymously considered with bedtime resistance, sleep anxiety, delayed sleep onset, and daytime sleepiness in the CSHQ, as shown in the appendices [D]. Therefore, the results of this study are consistent with those of previous studies.

The questions in subitem JSQP VIII address daytime behaviors, which involve restlessness and a lack of concentration during the daytime. In this study, 20 of the 45 children in the ASD group had ADHD as a comorbidity, whereas none of the children in the non‐ASD group did. Therefore, the result may be heavily influenced by ADHD symptoms. This finding is supported by the significant difference in the total score on the ADHD‐RS between the two groups in this study.

### Limitation

This study has several limitations. First, this study has the limitation of low power due to the small sample size. The fact that multiple regression analysis results could not be calculated in the non‐ASD group highlights this limitation. Future studies should include larger sample sizes.

Second, there are also limitations that blood tests are only once in the morning. Therefore, a delayed phase of the rhythm of melatonin levels is merely speculative. To verify this, studies involving multiple melatonin measurements are recommended.

Third, because this was a cross‐sectional study, it is not possible to determine a causal relationship between sleep problems and morning serum ferritin and melatonin levels.

Fourth, the analysis of confounding factors is insufficient. Although the children's blood sample collection time was standardized at 8:30, conditions such as children's bedtime and waking time, light exposure by ICT equipment, and sleep environment, such as light, sound, and temperature, which are potential confounding factors, were not standardized. In addition, the children's diets were not considered, although they could contribute to differences in the serum ferritin level. This is because these data were not sufficiently collected in the HFC study. Furthermore, in this study, control for IQ scores could not be performed because there were some children for whom IQ could not be calculated, while it is known that children with ID tend to have more sleep problems.^38^

## CONCLUSION

This study indicated that children with ASD were more likely to have some sleep problems and higher morning serum ferritin levels than those without ASD. However, the correlation between sleep problems and morning serum melatonin and ferritin levels was not clarified. Further research with a larger sample size is recommended.

## AUTHOR CONTRIBUTIONS

**Ai Terui:** Conceptualization; investigation; data curation; writing—original draft; funding acquisition. **Manabu Saito:** Conceptualization; supervision; project administration; writing—review and editing; funding acquisition. **Asami Kuki:** Methodology; writing—review and editing. **Shuji Shimoyama:** Investigation; writing—review and editing. **Yui Sakamoto:** Data curation; writing—review and editing. **Kazutaka Yoshida:** Data curation; writing—review and editing. **Ayako Osato:** Data curation; writing—review and editing. **Kazuhiko Nakamura:** Writing—review and editing; funding acquisition.

## CONFLICT OF INTEREST STATEMENT

The authors declare no conflicts of interest.

## ETHICS APPROVAL STATEMENT

This study was approved by the Ethics Committee of Hirosaki University Graduate School of Health Science (approval numbers 2013‐293, 2015‐055, 2016‐210, 2018‐168, 2018‐168‐2, 2018‐168‐3, and 2023‐033). The studies were conducted in accordance with local legislation and institutional requirements.

## PATIENT CONSENT STATEMENT

Written informed consent for participation in this study was provided by the children's legal guardians.

## CLINICAL TRIAL REGISTRATION

This study did not require registration as it was not a clinical trial.

## Supporting information

Appendices.

Appendices2.

## Data Availability

The raw data supporting the conclusions of this article will be made available by the authors, without undue reservation.
