# Supplementary material for: Correlation between sleep problems and morning serum melatonin and ferritin levels in Japanese 5‐year‐old children with autism spectrum disorder
Source: PCN Rep. 2026 Feb 10;5(1):e70294. doi: 10.1002/pcn5.70294 (PMC12887820; doi:10.1002/pcn5.70294)
Supplement: Supplementary file 1 — Appendices. [file PCN5-5-e70294-s001.docx]

**Appendices**

[A] Questionnaires and examinations used in the HFC study

| Table A.1 Questionnaires at the first phase of checkup | |
| --- | --- |
| ASSQ  (Autism Spectrum Screening Questionnaire) | Screening test for high-functioning ASD; 27-item, 3-point scale. Parents answer the questions in this study and are asked about social interaction, communication problems, limited interests and behaviors, or repetitive behavior problems [43][44]. |
| SDQ  (Strength and Difficulties Questionnaire) | Questionnaire that assesses children's emotions and behavior; 25-item, 3-point scale. It has four subscales of difficulties (emotional symptoms, conduct problems, hyperactivity/inattention, peer problems) and one subscale of strengths (prosocial behavior) [45]. |
| ADHD-RS-IV  (ADHD-Rating Scale Ⅳ) | A test to evaluate ADHD symptoms; 18-item, 4-point scale. Parents answer the questions in this study, which measures two characteristics of ADHD: inattention (9 items) and hyperactivity/impulsivity (9 items) [46]. |
| DCDQ  (Developmental Coordination Disorder Questionnaire) | Questionnaire test of motor coordination; 15-item, 5-point scale. Parents answer the questions. It has three subitems: control during movement, fine motor, and general coordination [47]. |
| PSI  (Parenting Stress Index) | Questionnaire test to measure parenting stress; 78-item, 5-point scale. Parents answer the questions. It consists of stress items related to the characteristics of the child (38 questions) and stress items related to the parents themselves (40 questions), and only stress items related to the characteristics of the child were administered in this study [48]. |

| Table A.2 Children who met at least one of the following criteria were considered at high risk for NDDs (screening positive). |
| --- |
| 1. Parent-rated ASSQ≥19 |
| 1. (ASSQ≧9) and [two or more items from (ADHD-RS-total≧M19/F14, ADHD-RS-inattention≧M12/F9, ADHD-RS-hyperactivity/impulsively≧M8/F5)] |
| 1. (SDQ-T≥ M18/F13)   and at least one of (ASSQ≥9, ADHD-RS-total≧M19/F14, ADHD-RS-inattention≧M12/F9, ADHD-RS-hyperactivity/impulsively≧M8/F5, DCDQ-fine motor and handwritings≦M8/F10, DCDQ-general coordination≦M11/F13) |
| 1. PSIC> 75%ile of PSI score distributions |

| Table A.3 Questionnaires and examinations at the second phase of checkup | |
| --- | --- |
| MABC-2  (Movement Assessment Battery for Children, 2nd edition) | A battery of tests to identify motor impairments, consisting of an 8-task motor test (covering the areas of manual dexterity, ball skills, and static and dynamic balance) and a checklist. No checklists were used in this study [49]. |
| WISC-Ⅳ  (Wechsler Intelligence Scale for Children - 4th Edition) | An intelligence test for 5 to 16-year-olds, and 12 tests are administered in this study. The FSIQ, Verbal Comprehension Index, Perceptual Reasoning Index, Working Memory Index, and Processing Speed Index scores are calculated. |
| Tanaka-Binet Intelligence Scale V | In the present study, this test was administered to children for whom the WISC-Ⅳ could not be administered; it is an intelligence test for children aged 2 years and older and was modified for Japanese. |
| Conners-3  （Conners 3rd Edition） | Questionnaire test to assess ADHD symptoms. Consists of 110 questions for parents and 115 questions for teachers. It includes components such as a primary factor scale (inattention, hyperactivity/impulsivity, learning problems, executive functioning, and challenge/aggression) and a symptom scale based on DSM-IV/5 (ADHD inattention, ADHD hyperactivity-impulsivity, predisposition, and defiance provocation).  We used this test as a diagnostic aid because the target age range is 6-18 years; 5-year-olds are not eligible. |
| DISCO  (Diagnostic Interview for Social and Communication Disorders 11th edition) | A semistructured interview-based diagnostic rating scale consisting of approximately 300 questions. In this study, only questions on the Early Childhood and NDD items were asked under the supervision of trained and qualified personnel [50]. |

[B] Questions of Japanese Sleep Questionnaire for Preschoolers (JSQP)

| Subitems | Questions |
| --- | --- |
| I. RLS sensory | Q14 says legs hurt at night |
|  | Q15 says legs feel hot at night |
|  | Q16 says legs feel strange at night |
| II. RLS motor | Q17 rubs feet at night |
|  | Q18 touches feet at night |
| III. OSAS | Q11 moves a lot during the night |
|  | Q29 clenches teeth |
|  | Q30 sleeps with mouth open |
|  | Q31 sleeps with head arched back |
|  | Q32 snores loudly |
|  | Q33 stops breathing |
|  | Q34 snorts and gasps |
| IV. Parasomnias | Q25 cries at night |
|  | Q26 wakes screaming and cannot be calmed down |
|  | Q27 woken by scary dreams |
|  | Q28 wakes up at any little sound |
|  | Q37 wakes more than once during the night |
| V. Insomnia or circadian rhythm disorder | Q4 late for nursery school or kindergarten due to waking up late |
|  | Q5 gets more than 2 naps |
|  | Q12 snoozes at nursery school or kindergarten |
|  | Q13 goes to bed after 10:00 PM |
|  | Q19 gets excited at night |
|  | Q20 gets grumpy at night |
|  | Q22 taken for a car ride due to sleeping difficulty |
|  | Q23 has trouble going to sleep |
|  | Q38 has no pattern to sleep and wakeup times |
|  | Q39 day to night reversal |
| VI. Morning symptoms | Q1 grumpy in the morning |
|  | Q2 needs a long time to wake up |
|  | Q3 takes a long time to get out of bed |
| VII. Daytime excessive sleepiness | Q6 falls asleep during the daytime |
|  | Q9 seems sleepy in the daytime |
|  | Q10 looks rundown in the daytime |
| VIII. Daytime behaviors | Q7 restless in the daytime |
|  | Q8 has poor concentration in the daytime |
| IX. Sleep habit | Q21 sleeps without being tucked in |
|  | Q24 goes to bed on his or her own |
| X. Insufficient sleep | Q35 stays up later than usual by an hour or more the day before a holiday |
|  | Q36 wakes up more than an hour later on a holiday |

[C] Examination of which subitem of the CSHQ applies to the question of the JSQP V: insomnia and circadian rhythm disorder.

| Question of JSQP V: insomnia or circadian rhythm disorder | Subitems considered applicable in CSHQ |
| --- | --- |
| ・late for nursery school or kindergarten due to waking up late | daytime sleepiness |
| ・gets more than 2 naps | daytime sleepiness |
| ・snoozes at nursery school or kindergarten | daytime sleepiness |
| ・goes to bed after 10:00 PM | bedtime resistance |
| ・gets excited at night | bedtime resistance |
| ・gets grumpy at night | sleep anxiety |
| ・taken for a car ride due to sleeping difficulty | night wakings |
| ・has trouble going to sleep | night wakings |
| ・has no pattern to sleep and wakeup times | bedtime resistance |
| ・day to night reversal | － |

[43] Ehlers S, Gillberg C, Wing L. A screening questionnaire for Asperger syndrome and other high-functioning autism spectrum disorders in school age children. *J Autism Dev Disord*. 1999;29(2):129-141. doi:10.1023/a:1023040610384

[44] Adachi M, Takahashi M, Takayanagi N, et al. Adaptation of the Autism Spectrum Screening Questionnaire (ASSQ) to preschool children [published correction appears in PLoS One. 2018 Aug 27;13(8):e0203254. doi: 10.1371/journal.pone.0203254.]. *PLoS One*. 2018;13(7):e0199590. Published 2018 Jul 10. doi:10.1371/journal.pone.0199590

[45] Goodman R. The Strengths and Difficulties Questionnaire: a research note. *J Child Psychol Psychiatry*. 1997;38(5):581-586. doi:10.1111/j.1469-7610.1997.tb01545.x

[46] DuPaul, George J, Power, Thomas J, Anastopoulos, Arthur D, et al. ADHD rating scale-IV : checklists, norms, and clinical interpretation, akashi shoten, Tokyo, 2016. in Japanese

[47] Nakai A, Miyachi T, Okada R, et al. Evaluation of the Japanese version of the Developmental Coordination Disorder Questionnaire as a screening tool for clumsiness of Japanese children. *Res Dev Disabil*. 2011;32(5):1615-1622. doi:10.1016/j.ridd.2011.02.012

[48] Narama M, Kanematsu Y, Araki A, et al. Validity and Reliability of the Japanese Version of the Parenting Stress Index. *J. Child Health*. 1999; 58(5):610-616. in Japanese.

[49] Hirata S, Kita Y, Yasunaga M, et al. Applicability of the Movement Assessment Battery for Children-Second Edition (MABC-2) for Japanese Children Aged 3-6 Years: A Preliminary Investigation Emphasizing Internal Consistency and Factorial Validity. *Front Psychol*. 2018;9:1452. Published 2018 Aug 31. doi:10.3389/fpsyg.2018.01452

[50] Wing L, Leekam SR, Libby SJ, Gould J, Larcombe M. The Diagnostic Interview for Social and Communication Disorders: background, inter-rater reliability and clinical use. *J Child Psychol Psychiatry*. 2002;43(3):307-325. doi:10.1111/1469-7610.00023
